# Supplementary material for: Minimal genome-wide human CRISPR-Cas9 library
Source: Genome Biol. 2021 Jan 21;22:40. doi: 10.1186/s13059-021-02268-4 (PMC7818936; doi:10.1186/s13059-021-02268-4)
Supplement: Supplementary file 1 — Additional file 1: Figure S1. Randomised selection of n sgRNAs per gene. ROC curve derived from previously defined sets of essential and non-essential genes [6]. Standardized partial area under the ROC curve (AROC) calculated per cell line over the range of maximum false discovery rate of 20%. AROCs are compared between down-sampled sgRNAs and all sgRNAs available for all the covered genes. 10 random sgRNAs permutations without replacement per cell line and per n guides were performed and AROCs mean values are plotted. AROC were calculated for each replicate of Project Score [13] (n = 663). Figure S2. MinLibCas9 sgRNA rank and selection flowchart and sgRNA metrics comparison. a, flowchart describing the sgRNA selection procedure. b, efficiency metrics of Project Score library sgRNAs - KS metric (Kolmogorov Smirnov test) comparison to non-targeting guides, JACKS scores [14], Rule Set 2 [9] scores, and FORECasT [21] predicted percentage of in frame deletions produced - plotted together with guides median fold-changes calculated across 663 samples. Spearman correlation coefficients are reported in the lower triangle of the grid. Plots in the diagonal represent the distribution of the respective metric. Figure S3. Down-sample analysis of top n sgRNAs ranked using KS and JACKS metrics. Combined score discards sgRNAs with a JACKS score outside the range of [0, 2] and then selects the top n sgRNAs according to the KS score (descending order, stronger KS scores and thereby stronger absolute fold-changes). Essential/Non-essential AROCs are the area under the ROC curve (at 20%FDR) using known essential and non-essential genes. Precision and recall rates are calculated between the sets of significant gene-level dependencies (at 1% FDR) estimated using the original library and the down-sampled library. Each box-and-whisker plot shows 1.5 x interquartile ranges and 5–95th percentiles, centres indicate medians (n = 245 cancer cell lines from Project Score [13]). Figure S4. MinLib [file 13059_2021_2268_MOESM1_ESM.pdf]

Supplementary figures

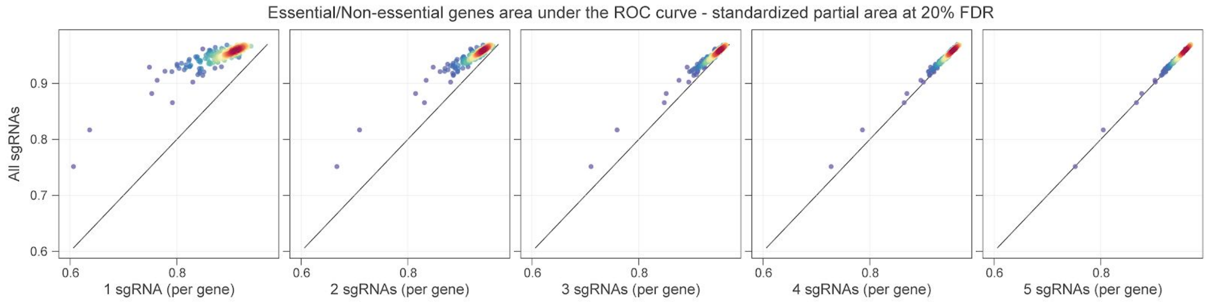

Fig S1. Randomised selection of n sgRNAs per gene. *ROC curve derived from previously defined sets of essential and non-essential genes [6]. Standardized partial area under the ROC curve (AROC) calculated per cell line over the range of maximum false discovery rate of 20%. AROCs are compared between down-sampled sgRNAs and all sgRNAs available for all the covered genes. 10 random sgRNAs permutations without replacement per cell line and per n guides were performed and AROCs mean values are plotted. AROC were calculated for each replicate of Project Score [13] (n=663).*

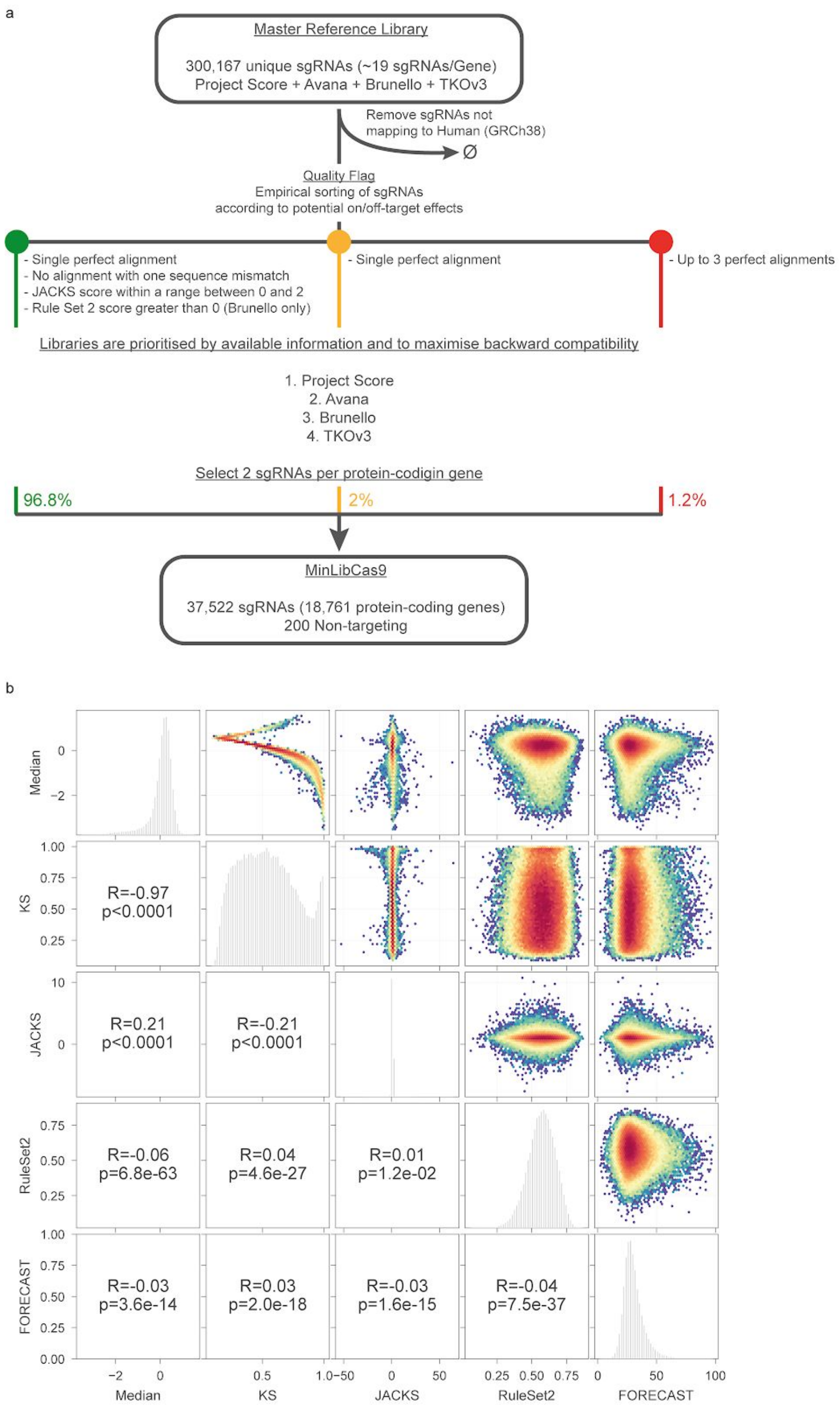

Fig S2. ***MinLibCas9 sgRNA rank and selection flowchart and sgRNA metrics comparison. a,*** *flowchart describing the sgRNA selection procedure.* ***b****, efficiency metrics of*

Project Score library sgRNAs - KS metric (Kolmogorov Smirnov test) comparison to non-targeting guides, JACKS scores [14], Rule Set 2 [9] scores, and FORECasT [21] predicted percentage of in frame deletions produced - plotted together with guides median fold-changes calculated across 663 samples. Spearman correlation coefficients are reported in the lower triangle of the grid. Plots in the diagonal represent the distribution of the respective metric.

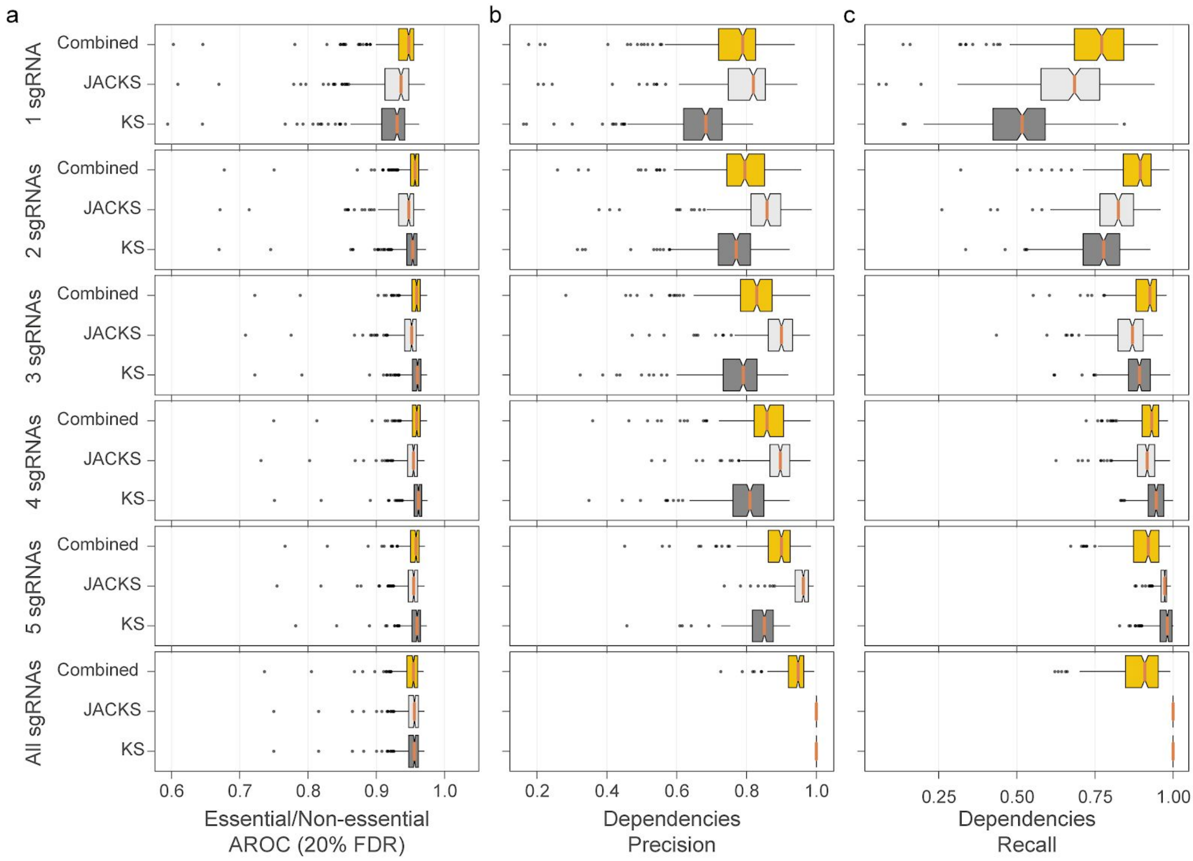

Figure S3. Down-sample analysis of top n sgRNAs ranked using KS and JACKS metrics. *Combined score discards sgRNAs with a JACKS score outside the range of [0, 2] and then selects the top n sgRNAs according to the KS score (descending order, stronger KS scores and thereby stronger absolute fold-changes). Essential/Non-essential AROCs are the area under the ROC curve (at 20%FDR) using known essential and non-essential genes. Precision and recall rates are calculated between the sets of significant gene-level dependencies (at 1% FDR) estimated using the original library and the down-sampled library. Each box-and-whisker plot shows 1.5 x interquartile ranges and 5–95th percentiles, centres indicate medians (n=245 cancer cell lines from Project Score [13]).*

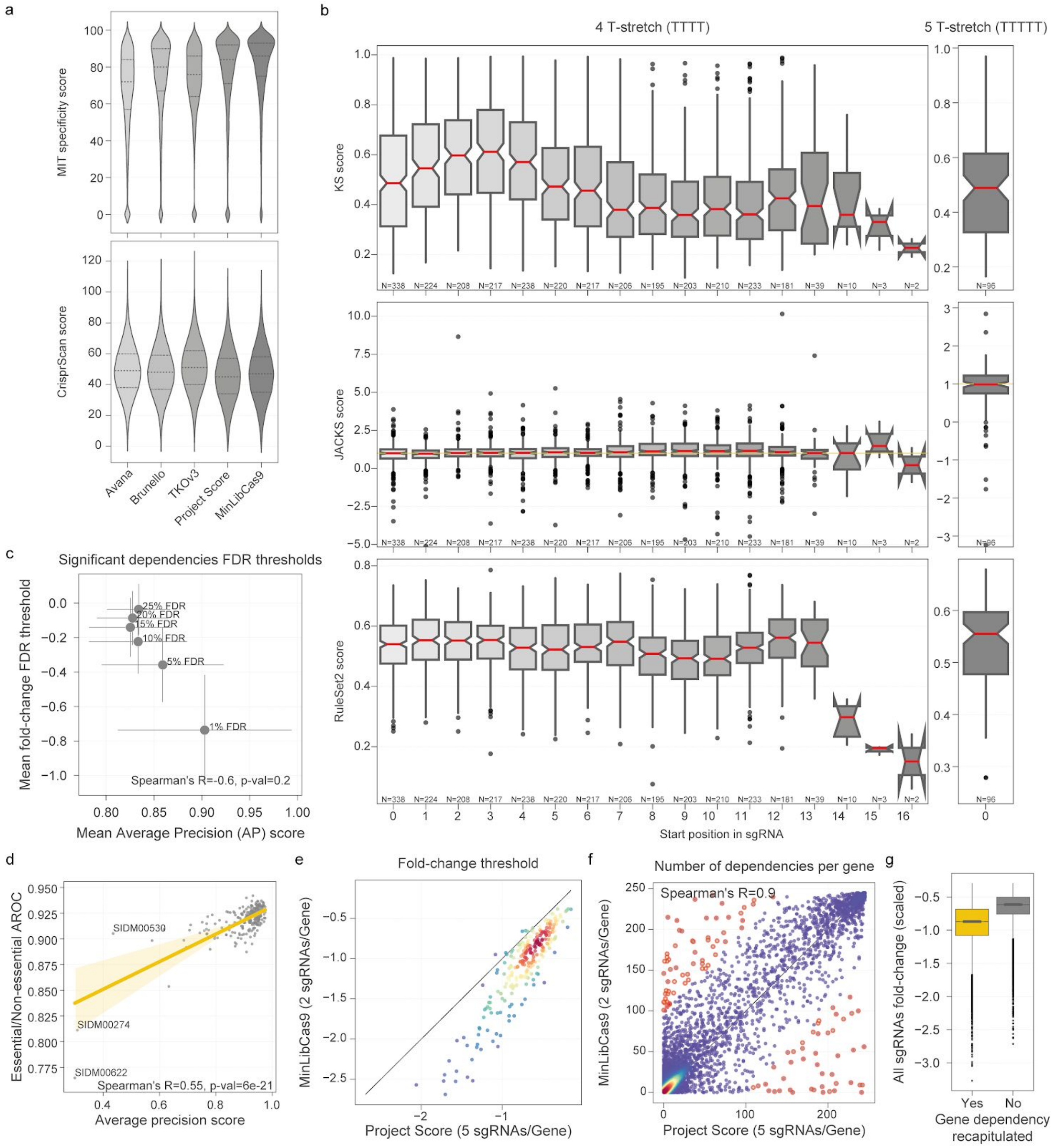

Fig S4. MinLibCas9 benchmarking. ***a****, distributions of CRISPOR sgRNA scores.* ***b****, distributions of KS, JACKS and RuleSet2 scores for sgRNAs from Project Score library that contain T-stretches of 4 and 5.* ***c****, significant dependencies identified using different FDR rates between essential and nonessential genes (1%, 5%, 10%, 15%, 20% and 25%). MinLibCas9 fold-change thresholds at each FDR rate and the average precision (AP) of the gene dependencies identified in MinLibCas9 that are also found with Project Score library. Error bars represent standard deviation.* ***d****, average precision scores per cell line of established dependencies with MinLibCas9 fold-changes, calculated through downsampling the original screens, correlated with Project Score data quality as measured by the area*

under the ROC curve (AROC) using known essential and non essential genes. ***e***, *MinLibCas9 fold-change threshold for each cell line identified at 1% FDR of essential versus non-essential genes plotted against those identified with Project Score library . Colour represents density.* ***f***, *cumulative number of dependencies (at 1% FDR) identified in 245 cancer cell lines for each gene with both the full original library and the minimal library.* ***g***, *scaled fold-changes (median essential genes fold-change = 1; median non-essential genes fold-change = 0) of dependencies recapitulated (n=251,387) and missed (n=36,996) with the minimal library (two-sided Welch's t-test p-value < 0.001). Each box-and-whisker plot shows 1.5 x interquartile ranges and 5–95th percentiles, centres indicate medians.*

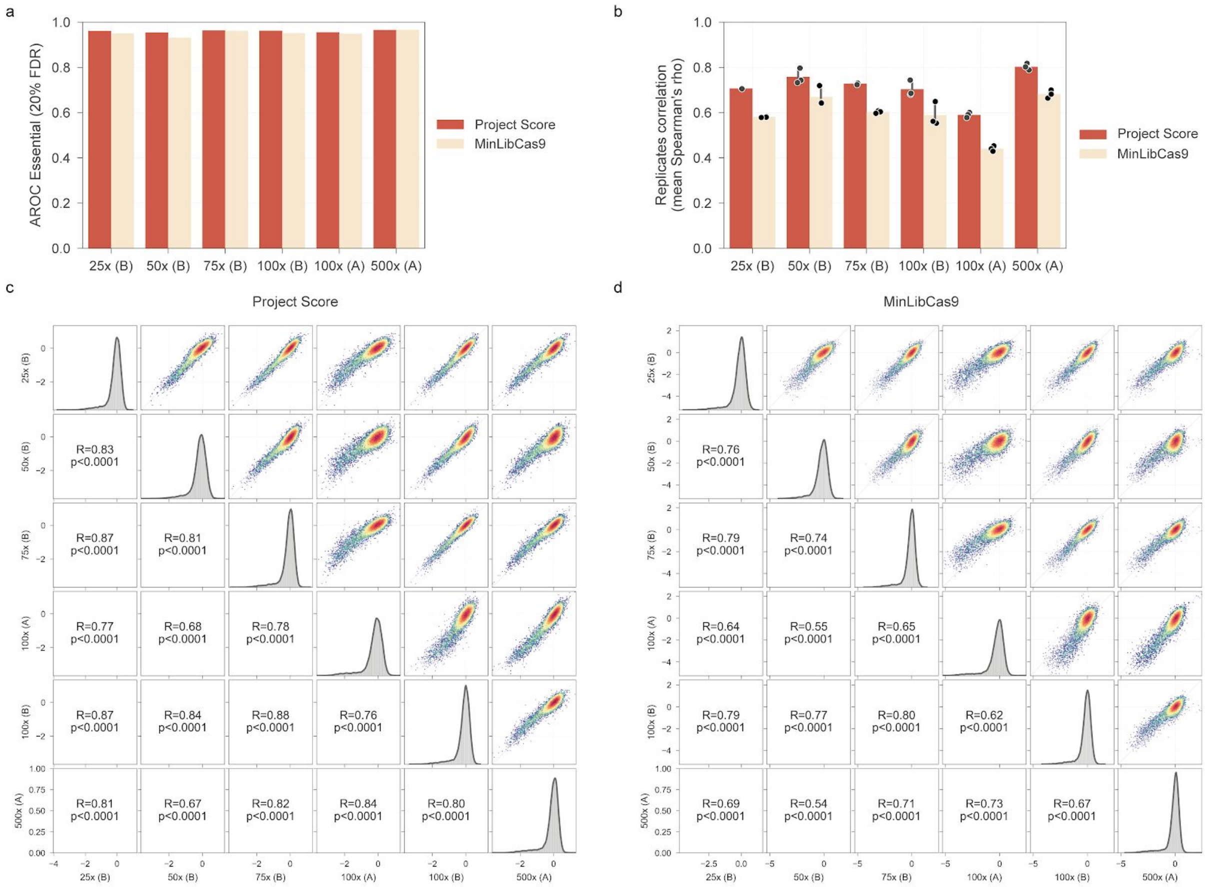

Fig S5. sgRNA library coverage analysis in KM-12 cancer cells. a, *AROC of essential/non-essential genes at different guide coverage levels.* ***b***, *technical replicates correlation. Comparisons are made between the original Project Score library and the in silico minimal library.* ***c*** *and* ***d***, *show correlation plots between the different library coverage for Project Score and downsampled MinLibCas9 libraries, respectively. A and B represent two independent experiments.*

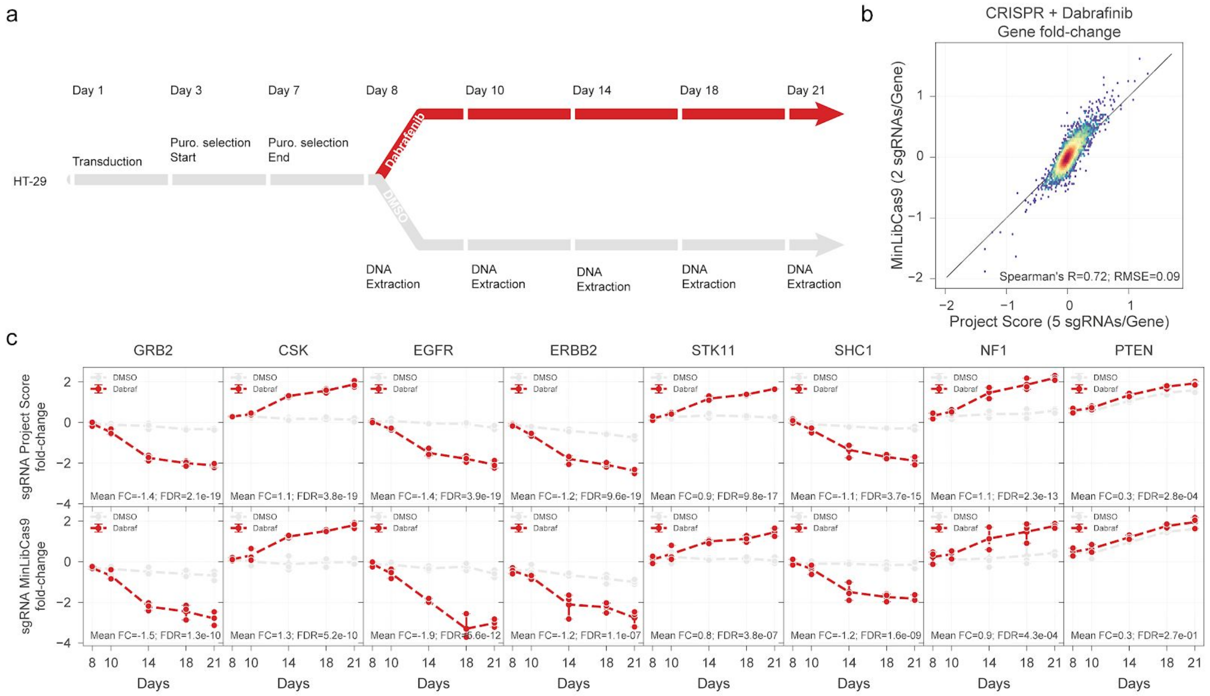

Fig S6. CRISPR-Cas9 dropout screens upon treatment with Dabrafenib. a, *diagram of the experimental setup.* ***b***, *gene fold-changes averaged across the different time points (day 8, 10, 14, 18 and 21) obtained using Project Score library compared to the in silico MinLibCas9. Colour represents point density.* ***c***, *time-series fold-changes of the top significantly essential hits (compared to control experimental arm, DMSO) obtained with Project Score and MinLibCas9 library, the values of three technical replicates are represented with the mean and by the minimum and maximum values as error bars.*

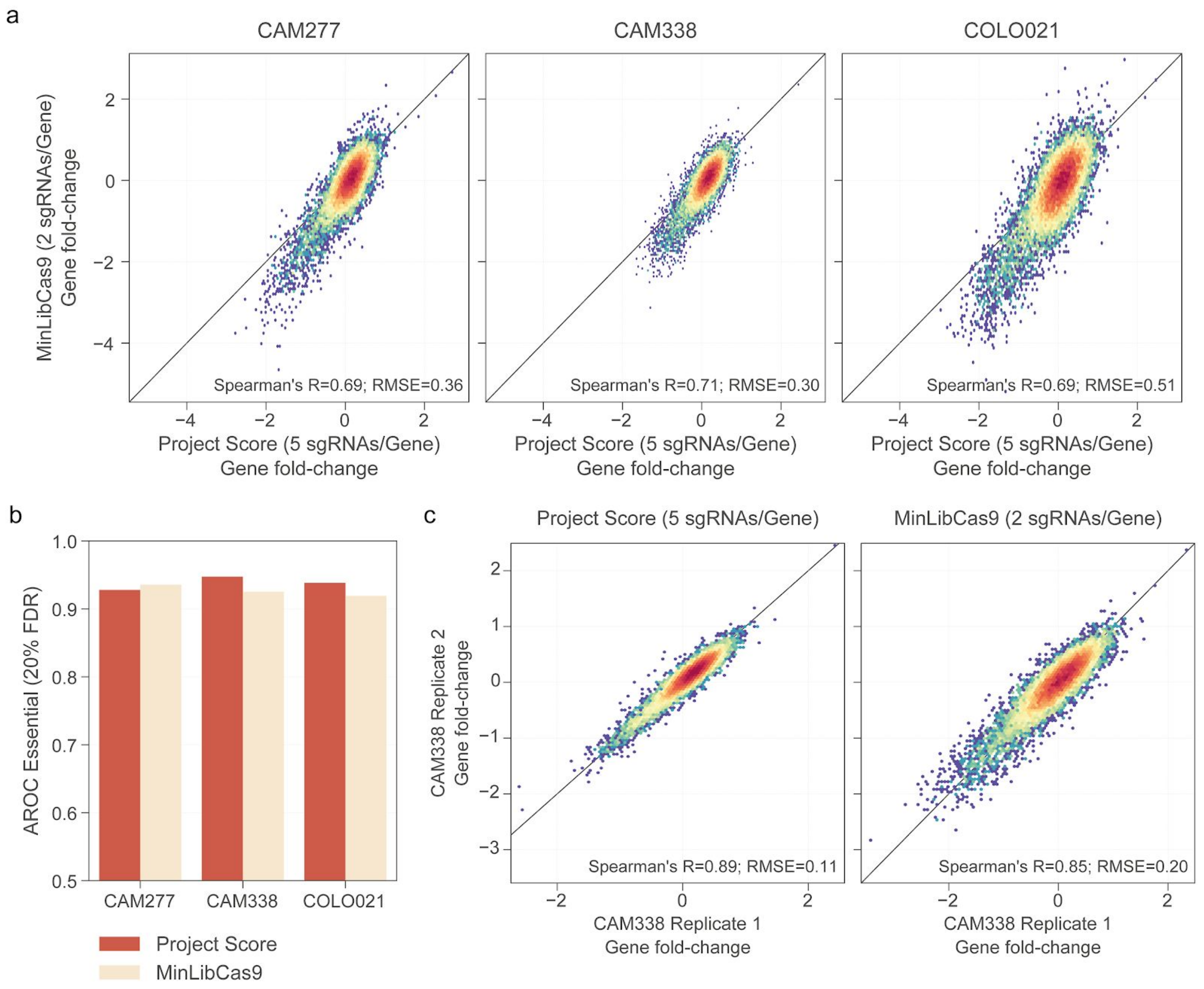

Fig S7. CRISPR-Cas9 loss-of-fitness screens in 3D organoids. a, *Comparison of gene fold-changes obtained using the in silico minimal and original library in a colon carcinoma organoid (COLO021) and two oesophageal adenocarcinoma organoids (CAM277 and CAM338).* ***b***, *AROC of essential/non-essential genes of each organoids obtained with both libraries.* ***c***, *CAM338 technical replicates correlation. Colour coding represents point density.*

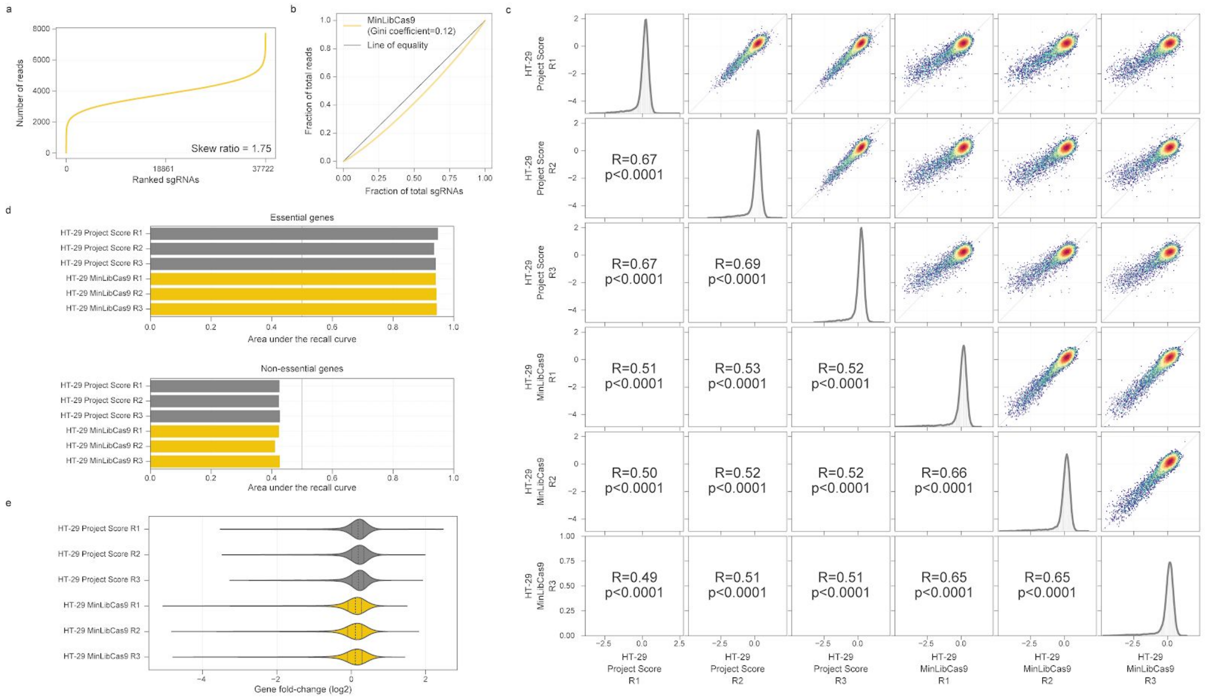

***Fig S8. MinLibCas9 screens in HT-29 cancer cell line. a**, Distribution of guides within MinLibCas9 library plasmid calculated to be comparatively even. Skew ratio of top 10% vs bottom 10% guides = 1.75 (minimum read count = 30, maximum read count 7698). **b**, lorenz curve of the MinLibCas9 plasmid library, Gini coefficient = 0.12. **c**, correlation of gene level fold-changes of the three technical replicates of MinLibCas9 screens versus the three technical replicates of HT-29 screens performed in Project Score. Spearman's Rho correlations are reported on the lower diagonal, fold-changes distributions are displayed in the diagonal, and upper diagonal shows the scatter plots between the two samples fold-changes colored by density. **d**, recall of previously defined essential (upper panel) and non-essential (lower panel) genes quantified by the area under the recall curve. **e**, distributions of gene level fold-changes for each sample. Box-and-whisker plot shows 1.5 x interquartile ranges and 5–95th percentiles, centres indicate medians.*

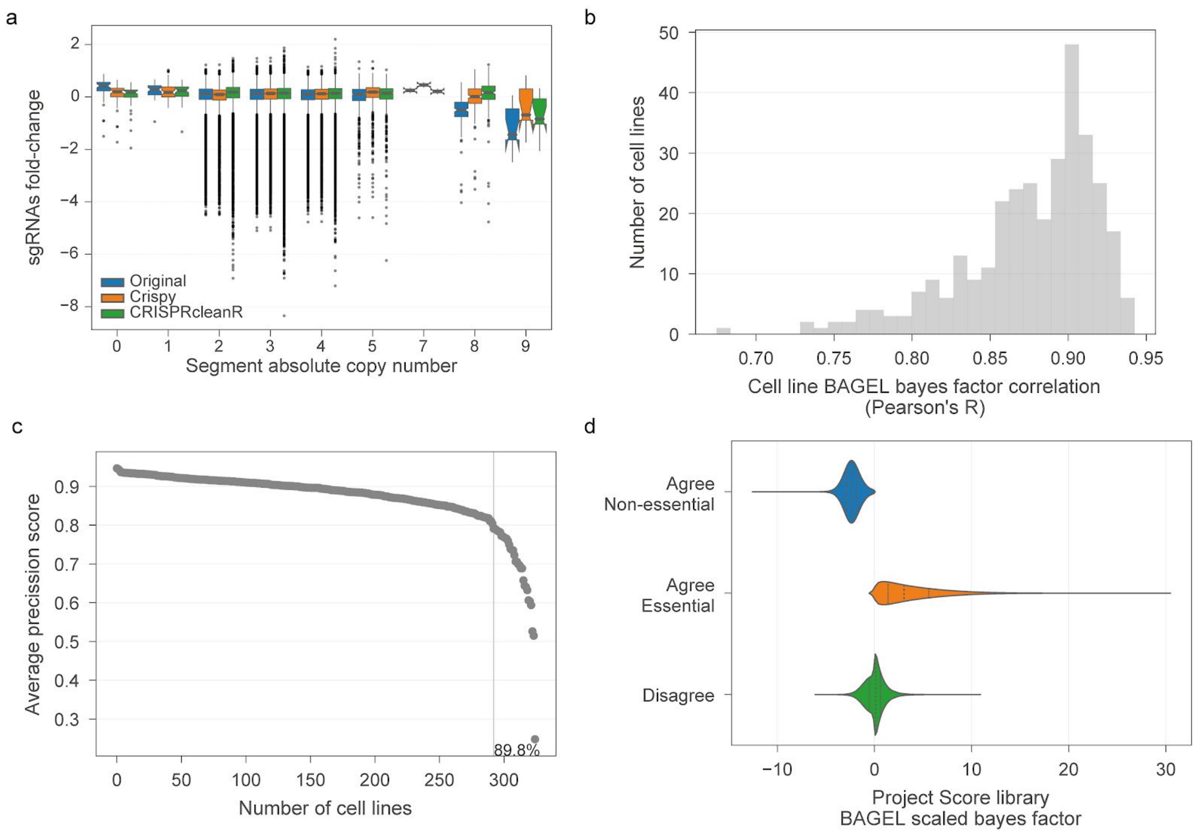

***Fig S9. Application of common CRISPR-Cas9 analytical pipelines to MinLibCas9. a**, sgRNA fold-change distributions of HT-29 MinLibCas9 screens before (Original) and after copy number bias correction using supervised (Crispy [28]) and unsupervised (CRISPRcleanR [34]) approaches across the different levels of copy number amplifications. **b**, distribution of the correlation coefficients between the downsample MinLibCas9 and Project Score BAGEL bayesian factors across Project Score CRISPR-Cas9 screens (n=325). **c**, average precision (AP) scores of the precision-recall curves of BAGEL significantly essential genes from Project Score (at 5% FDR) obtained considering MinLibCas9 BAGEL bayesian factors as rank classifier (one point per cell line). Vertical line represents the percentage of cell lines with at least 80% AP score. **d**, distributions of the BAGEL bayesian scores calculated with the Project Score for the genes found to be non-essential with both Project Score and downsample MinLibCas9 (Agree Non-essential), essential with both libraries (Agree Essential), and that are essential in only one library (Disagree).*
